# Abundance of Lipopolysaccharide Heptosyltransferase I in Human Gut Microbiome and Its Association With Cardiovascular Disease and Liver Cirrhosis

**DOI:** 10.3389/fmicb.2021.756976

**Published:** 2021-11-30

**Authors:** Shujin Lin, Hui Zhang, Xueke Wang, Ting Lin, Zihan Chen, Jingfeng Liu, Jianmin Wang

**Affiliations:** ^1^Fujian Cancer Hospital, Fujian Medical University Cancer Hospital, Fuzhou, China; ^2^College of Biological Science and Engineering, Fuzhou University, Fuzhou, China

**Keywords:** gut microbiota, lipopolysaccharide, heptosyltransferase, health, disease

## Abstract

Lipopolysaccharide (LPS) is a potent endotoxin on the outer membrane of gram-negative bacteria. Heptosyltransferase I (HpeI) takes part in the synthesis of LPS. In this study, we first collected the protein sequences of HpeI homologs from the human microbiome. The collected HpeI sequences was classified based on sequence similarity, and seven clusters of HpeI were obtained. Among these clusters, proteins from Cluster 3 were abundant in the human mouth, while Clusters 1, 6, and 7 were abundant in the human gut. In addition, proteins from Cluster 1 were mainly from the order of Enterobacterales, while Cluster 6 and 7 were from Burkholderiales. The correlation analysis indicated that the total abundance of HpeIs was increased in patients with cardiovascular disease and liver cirrhosis, and HpeI in Cluster 1 contributed to this increase. These data suggest that HpeI homologs in Cluster 1 can be recognized as biomarkers for cardiovascular disease and liver cirrhosis, and that reducing the bacterial load in Cluster 1 may contribute to disease therapy.

## Introduction

Lipopolysaccharide (LPS), a biomolecule component, is abundant on the bacterial cell surface and is critical to the resistance of bacterial cells to environmental stress. LPS is also an important endotoxin found in bacterial infections of the intestines, gums, skin, and other tissues (Raetz and Whitfield, 2002). It is a pathogen-associated molecular pattern (PAMP) molecule composed of three structural domains: hydrophilic polysaccharides or oligosaccharide core, O-antigen, and lipophilic lipid A (immunostimulatory component) (Rosenfeld and Shai, 2006). In the human gut, LPS is primarily derived from Bacteroidales, and an immunosuppressive effect exists in the total LPS in the intestinal tract of adults (d’Hennezel et al., 2017). Injecting LPS into animals can cause fever as it is a pyrogenic substance. Since there are fewer bacteria that produce a lot of LPS, the amount of LPS in the intestinal tract may be low. However, bacterial LPS was found to be involved in the occurrence and development of many diseases such as chronic inflammation of the intestines, liver damage, diabetes, Alzheimer’s, and Parkinson’s disease (Gomes et al., 2017; Wassenaar and Zimmermann, 2018). By optimally controlling intestinal LPS activity, local intestinal integrity and systemic host homeostasis may be maintained. Proteus produces a proto-inflammatory LPS, which can induce a strong inflammatory response, causing septic shock and even death. On the other hand, bacteria such as Bacteroides produce an anti-inflammatory LPS, which exhibits antagonistic activity in response to a pro-inflammatory response (Lin et al., 2020).

The biosynthesis of LPS is triggered by the continuous addition of sugar moieties. The formation of the LPS core region depends on the process of adding multiple heptose sugars catalyzed by heptatransferase (Hep). Currently, four types of Heps are found in all gram-negative bacteria (Cote and Taylor, 2017). In the process of adding the first two sugars to the catalytic core, HepI and HepII always exist, while HepIII and HepIV are only identified in certain species (Reeves and Wang, 2002). The structure of HepI–IV are very similar as they all contain glycosyltransferase structure folds (Grizot et al., 2006). Cells lacking HepI show truncated LPS, which makes them more susceptible to hydrophobic antibiotics (Coleman and Leive, 1979). A recent study confirmed the interaction between LPS and the antibiotic during antibiotics transport at single molecule level (Wang J. et al., 2020).

HepI transfers the heptosyl unit from ADP-L-glycerol-β-D-mannose-heptose to the fifth position of Kdo2-lipid A. Therefore, highly truncated gram-negative bacteria may be caused by the inhibition of HepI, causing them to respond to the complement system, cationic antimicrobial peptides, vaccine-derived antibodies, phagocytosis, and the innate immune system (Wang et al., 2015; Kong et al., 2016; Blaukopf et al., 2018). Considering the importance of HepI, we analyzed the abundance of it in the human gut microbiome. We further investigated the relationship of the abundance of HpeI with diseases, which showed that HepI abundance is highly associated with **cardiovascular disease (CVD) and liver cirrhosis (LC).**

## Materials and Methods

### Sequence Information of HpeIs in Human Intestinal Microflora

The HpeI sequences were retrieved from the InterPro database (version: 86) and the results belonging to the human intestinal microflora were retained for further analysis (Blum et al., 2020). According to the above method, the sequence similarity network (SSN) and phylogenetic tree of HpeIs in the intestinal microflora were constructed (Zallot et al., 2019).

### Using Healthy Human Intestinal Metagenome Data to Determine HpeI Gene Abundance

According to previous reports, Short, Better Representative Extract Dataset (ShortBRED) was used to analyze the abundance of HpeI genes (Blum et al., 2020). Briefly, ShortBRED-Identify found peptide markers of the HpeI cluster, and the comprehensive protein reference catalog is UniRef90. After obtaining the markers, we used ShortBRED-Quantify to count the relative abundance. The study was conducted on the website^1^. Finally, the Reads per kilo base per million mapped reads (RPKM) values were converted into copies of each microbial genome (Levin et al., 2017).

### Analysis of the Abundance of HpeI Gene in Human Intestinal Metagenomics Under Disease Conditions

The dataset of human fecal metachondrial body related to diet were downloaded from the Sequence Read Archive (SRA) of NCBI. Only information from non-antibiotic/probiotic-treated cases and the use of the Illumina sequencing platform were included in this study. In NCBI’s SRA, the whole genome sequencing dataset was downloaded, which was about disease-related human fecal metagenomes (Supplementary Table 1). The host and data selected for analysis were from non-antibiotic/probiotic treatment and Illumina sequencing platforms, respectively. Quality trimming was performed using Sickle software,^2^ and the quality threshold and minimum lengths was 30 bp to homogenize the different data sets. The remaining sequencing reads were of high quality and were mapped to the nucleotide sequences of HpeIs that we have previously reported using the Burrows-Wheeler Alignment-Maximal Exact Matches (BWA-MEM) algorithm (Li and Durbin, 2009). The SAMTools is applied to remove results that showed mapping quality number (MAPQ) that is greater than 60 (Li et al., 2009). The number of reads is calculated by BEDtools.^3^ The taxonomic classification read was recognized by the Kaiju program (Menzel et al., 2016). The read counts of HpeIs were standardized and visualized by the R programming language.

### Statistical Analysis

R (version 3.6.1) was used for the statistical analysis. The normality of HpeI abundance was evaluated using the Shapiro–Wilk test. The analysis of the difference in HpeI abundance between the test subjects and the control group was performed using the paired Wilcoxon signed-rank test because the results did not have a normal distribution (Wirbel et al., 2019).

## Results

### Clustering and Phylogenetic Tree of Gut HepI

**FIGURE 1:**
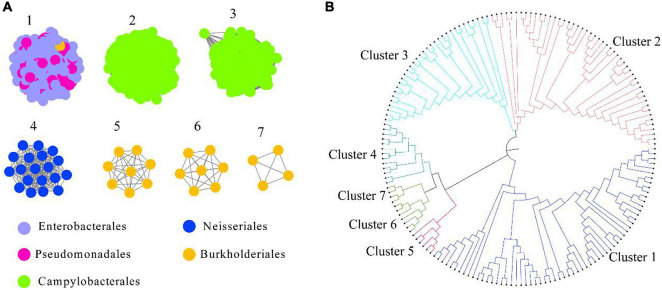
Intestinal HepI clustering based on sequence similarity network and phylogenetic tree. **(A)** HepI from the human gut microbiome analyzed to create the network (*e*-value < 10^–3^, sequence identity > 45%). The results showed 1,613 nodes and one node represents one protein sequence. Nodes from the same order paint the same color in the network. **(B)** Evolutionary relationships of HepIs in different clusters. Maximum-likelihood phylogenetic tree for the selected HepIs from each clusters were generated using MEGA 7.0.

HepI was placed in the InterPro consortium with accession number IPR011908. We collected all the protein sequences from IPR011908 using InterPro 86 (June 3, 2021). The sequences from the bacteria originating from the human microbiome was further collected, and a total of 1613 sequences were obtained (Supplementary Dataset 1). As shown in Figure 1A and Supplementary Figure 1, a criterion of 45% sequence identity was used to generate the SSN of intestinal HepI, which separated the 1613 sequences into seven clusters. The criterion was chosen as the classification of the proteins that fit the bacteria origin at the order level of taxonomy. The sequences were clustered together with 40% sequence identity. On the other hand, the sequences were separated into 10 clusters with 50% sequence identity. The first cluster among the seven clusters using 45% sequence identity contained sequences from Enterobacterales and Pseudomonadales. The second and third clusters were from Campylobacterales, while the sequences in the second cluster was from *Helicobacter.* The proteins in the third cluster were from *Campylobacter*. The fourth cluster comprised proteins from the order of Neisseriales. The proteins in the fifth, sixth, and seventh clusters were all from Burkholderiales. As a result of the phylogenetic analysis, the fourth, fifth, sixth, and seventh clusters formed a clade that was close to cluster 1. The proteins from clusters 2 and 3 form two other clades (Figure 1B).

### Abundance of HepI in Healthy Cases

**FIGURE 2:**
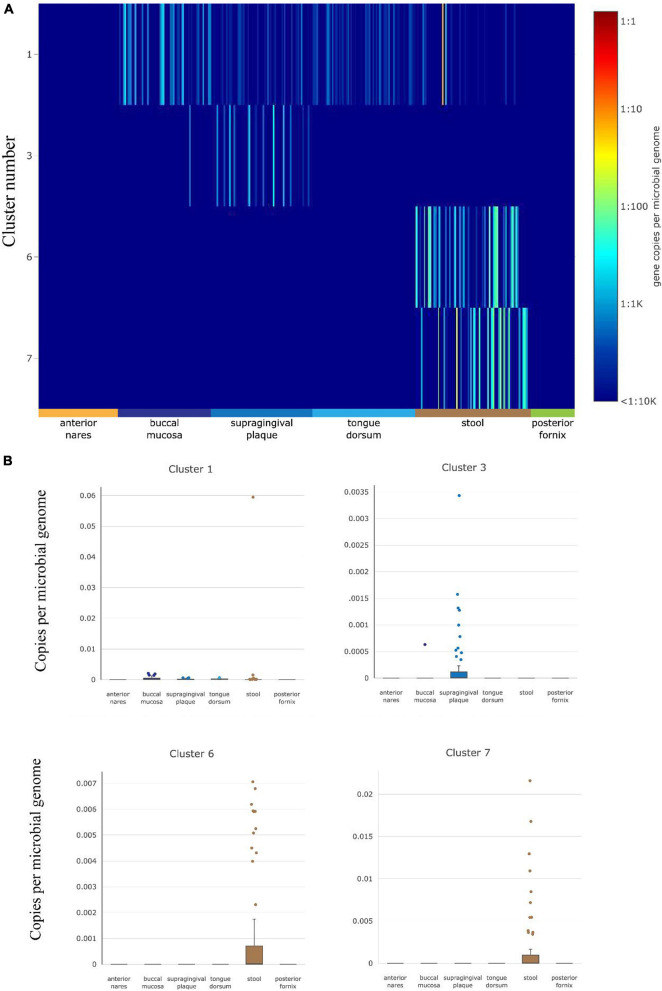
HepI abundance in the human microbiome datasets of healthy subjects. **(A)** Heatmap of the distribution and abundance of HepI clusters analyzed by ShortBRED, which include vaginal fornix, stool and facial parts such as the buccal mucosa, anterior nares, tongue dorsum. **(B)** Boxplots of clusters 1, 6, and 7 across different body sites.

The abundance of gut HepI in 380 metagenomes was studied using ShortBRED in healthy cases (Figure 2). Data were obtained from different human body sites: vaginal fornix, stool, and facial parts such as the buccal mucosa and anterior nares. Only HepIs from clusters 1, 3, 6, and 7 in the SSN were found in the healthy group. Enzymes from Cluster 1 were the most widely distributed in the human microbiome, including the buccal mucosa and stool. Cluster 3 was also found in supragingival plaques with a relatively high amount. In contrast, Clusters 6 and 7 were highly abundant only in the stool.

### HepIs Abundance in the Human Gut Under Disease Conditions

**FIGURE 3:**
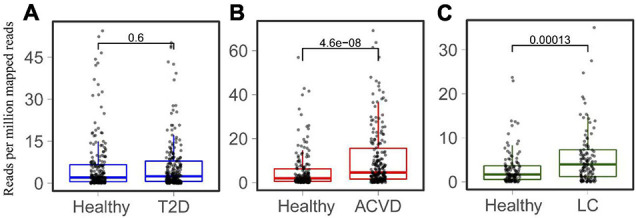
Comparison of the total abundance of HepIs in the human gut microbiome between a healthy group and a disease group. The metagenomic datasets are from the disease conditions; Type 2 diabetes T2D **(A)**, cardiovascular disease CVD **(B)**, and liver cirrhosis LC **(C)**. The paired Wilcoxon test was used to calculate the *p-*value for significant analysis.

Considering that LPS is associated with diabetes and liver-related diseases (Su, 2002; Bala et al., 2011; Pastori et al., 2017), the relationship between HepI abundance and diseases was studied. Metagenomic sequencing datasets linking human health with Type 2 diabetes (T2D), CVD, and LC were collected (Supplementary Table 1). The T2D datasets were obtained from both China and Sweden (*n* = 240, *n* = 226 CTRLs). The datasets of CVD and LC were all from China (*n* = 214, *n* = 171 CTRLs for CVD, *n* = 123, *n* = 114 CTRLs for LC). We mapped genes to the intestinal metagenomic datasets to quantify the abundance of the total HepI genes and calculated the significance of their value (Figure 3). The results showed that there was no significant difference in HepI abundance between healthy participants and T2D patients. However, HepI abundance in the CVD and LC subjects was significantly higher than that in the healthy controls (*p* = 4.6e-8 and *p* = 0.00013, respectively). Based on these analyses, we found that the abundance of HepI genes in the gut was positively related to CVD and LC. We further analyzed the taxonomic distribution of HpeIs in the cohorts. The data showed that HepIs in the gut are mainly from the order of Enterobacterales and Burkholderiales (Supplementary Figure 2). Comparison of the abundance of HpeIs in the T2D datasets showed that both abundances of HpeI in Enterobacterales and Burkholderiales did not show significant difference between healthy participants and T2D patients. However, the abundance of HpeI in Enterobacterales in the patients of CVD and LC increased significantly (*p* = 2.8e-10 and *p* = 9.5e-4, respectively). This data suggested that HpeI in Enterobacterales is much closely associated with the two disease conditions.

### The Relationship Between the Difference Cluster of Gut HepIs and Human Diseases

**FIGURE 4:**
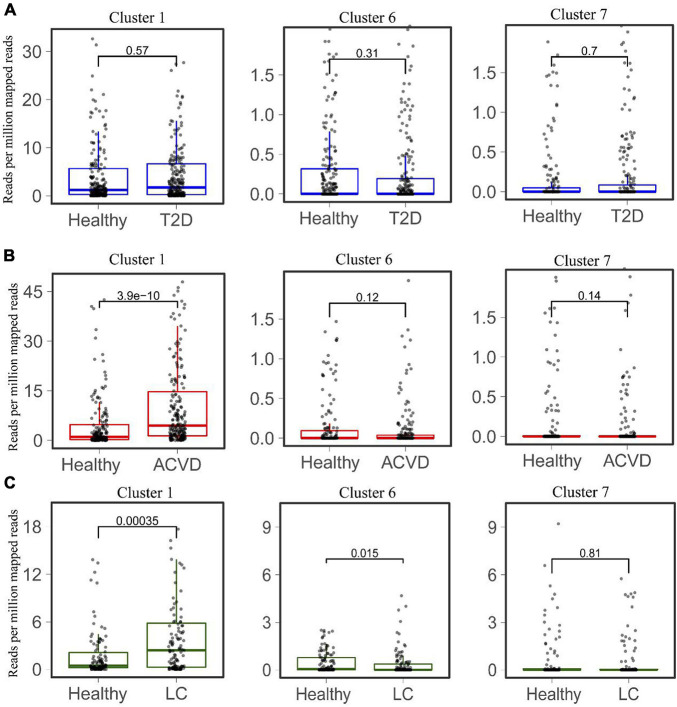
Comparison of the abundance of HepIs from different clusters in the human gut microbiome between a healthy group and a disease group. The metagenomic datasets from the disease conditions; Type 2 diabetes T2D **(A)**, cardiovascular disease CVD **(B)**, and liver cirrhosis LC **(C)** are used to map the genes of HepIs from Cluster 1, 6, and 7. The paired Wilcoxon test was used to calculate the *p*-value for significant analysis.

The distribution and abundance of Clusters 1, 6, and 7 in the healthy intestinal metagenomes indicated that the HepI in different clusters might play different roles in certain disease states. We then mapped the HepI in Clusters 1, 6, and 7 to the datasets mentioned above (Figure 4). Similar to the difference in the total abundance of HepI in T2D datasets, the abundance of HepI in Clusters 1, 6, and 7 between the healthy controls and T2D participants did not show any significance. In the case of CVD, the HepI in Cluster 1 increased significantly in the patients compared with the healthy participants (*p* = 3.9e-10), and there was no significant difference in the HepI in Clusters 6 and 7 between healthy and CVD subjects. The HepI in Cluster 1 in the patients with LC also showed a significant increase in comparison with the controls. In addition, the HepI in Clusters 6 and 7 in the dataset did not show any significant differences. These results suggested that the abundance difference of HepI in the gut between the healthy participants and CVD/LC patients was mainly due to the enzymes from Cluster 1.

## Discussion

Intestinal microbial LPS is not only considered to be one of the most effective factors for activating innate immune signal transduction, but is also considered as an important medium for the microbiome affecting host physiology (d’Hennezel et al., 2017). LPS from *E. coli* increases gut tight junction permeability and intestinal inflammation in a Toll-like receptors 4 (TLR4)-related manner, which is regulated by the activation of the focal adhesion kinase 1 signaling pathway (Guo et al., 2015). However, the capacity to trigger the activation is structure-dependent, which determines whether LPS is classified as an antagonist or an agonist (Munford, 2008; Di Lorenzo et al., 2019). Lipid A with bis-phosphorylated hexa-acylated motifs (such as *E. coli* lipid A) acts as an agonist of the TLR4/myeloid differentiation factor 2 complex and has a certain function of immune stimulation (Di Lorenzo et al., 2017). Next, few LPS compounds could bind TLR4 and compete with toxic LPS with an antagonistic property, thus preventing downstream inflammatory responses (Di Lorenzo et al., 2017). The structure of hexa-acylated LPS from *E. coli* is distinct from penta- and tetra-acylated lipid A of *Bacteroides*, which are the dominant species in the human gut microbiota. Our study shows that HepI can be found in Enterobacteriaceae, but not Bacteroides. This suggests that HepI may be one of the factors affecting the structure of LPS.

LPS can promote lipid accumulation in adventitial fibroblasts of humans and increase oxidative stress in atherosclerotic lesions. It can further increase the mortality and morbidity of atherosclerosis-related cardiovascular disease (Wang et al., 2012). Inflammation and oxidative stress caused by LPS also results in acute liver injury and failure (Jiang et al., 2018). Our data indicate that HepIs located in Cluster 1 are significantly related to both CVD and LC, suggesting that these enzymes and the bacteria encoding the enzymes can be used as potential markers for these diseases. Furthermore, inhibition of enzyme activity or reduction of bacterial load in the gut can be considered as an option to treat related diseases. The study to screen and design the inhibitors of HepIs has been performed and showed application potential for antimicrobial development (Wang A. et al., 2020).

Dietary factors may be related the abundance of HpeI and LPS in the human gut. It has been reported that dietary style, such as Western diet, high calorie diet, high fat diet (HFD), may increase the LPS abundance in both human and mice (Fuke et al., 2019; Bai et al., 2020). On the other hand, high-grain diet, probiotics, prebiotics, and polyphenols could reduce the level of LPS in goat liver (Chang et al., 2015; Fuke et al., 2019). In addition, HFD markedly alters the composition of microbiota with enriching the orders *Enterobacterales* (Calibasi-Kocal et al., 2021). Taken together, these studies suggested HFD-induced increase of *Enterobacterales* may produce more LPS synthesized by HpeI, and inhibition of HpeI increase and activity could contribute the related diseases.

Our data suggested that HpeI-encoding Enterobacterales increased in the gut of the patients with LC. Other studies showed that *E. coli*, *Klebsiella pneumoniae*, and other Enterobacterales are the most common and are responsible for up to 50% in patients with cirrhosis (Fernández et al., 2021). While both bacteria from Enterobacterales and Burkholderiales increased in the gut of CVD patients. These studies are also consistent with our conclusion and suggested that the increased bacteria, LPS, and HpeIs can be used as biomarker for the diseases. However, gene transfer occurs frequently in the gut microbiome (Groussin et al., 2021), suggesting biomarker based on bacteria species may not reflect the real function of strains. While the structure of the LPS is also very complex and the physiology function of each LPS may be diverse. Considering the specificity of enzymes to catalyze the reactions in the gut, we suggested that classified enzymes catalyzing different reactions are potentially precise biomarker for diseases.

The relationship between the abundance of microbiota in the gut and the diseases has been widely studied. Compared with the research analyzing the abundance of microbiota, relatively few studies between the functional genes from gut microbes and the diseases are performed. In the present study, we set up the relationship between the abundance of microbial HpeIs and the diseases, including CVD and LC. However, there are some limitations of the study. First, HpeIs might be also related to other diseases, such inflammation, immune disorders, neurological diseases, etc., considering the importance and diversity function of LPSs. Further study should be performed to analyze the association between HpeIs and the diseases. Second, the HpeIs in different clusters in the study might catalyze different reactions and form different structures of LPS. Thus, further experiments should be performed to confirm the function of HpeIs.

In conclusion, microbial LPSs in the human gut have considered as an endotoxin, while HpeI is the key enzyme catalyzing the synthesis of LPS. In the present study, we collected the sequences of HpeI from gut microbiota and separated them into 7 clusters. The HpeIs in the first cluster showed the highest amount in the human gut, which were mainly from Enterobacterales. Further study showed that the abundance of HpeIs in the Cluster 1 was positive associated with the CVD and LC. On the basis of the results, we proposed that the HpeIs in the Cluster 1 could be considered as a biomarker and therapy target for the diseases.

## Data Availability Statement

The datasets presented in this study can be found in online repositories. The names of the repository/repositories and accession number(s) can be found in the article/Supplementary Material.

## Author Contributions

JL, JW, and SL designed the project, performed evolution analysis, wrote the draft manuscript, supervised the experiment, and wrote the manuscript. HZ and XW collected metagenome datasets and processed raw data. TL and ZC helped data analysis. All authors contributed to the article and approved the submitted version.

## Conflict of Interest

The authors declare that the research was conducted in the absence of any commercial or financial relationships that could be construed as a potential conflict of interest.

## Publisher’s Note

All claims expressed in this article are solely those of the authors and do not necessarily represent those of their affiliated organizations, or those of the publisher, the editors and the reviewers. Any product that may be evaluated in this article, or claim that may be made by its manufacturer, is not guaranteed or endorsed by the publisher.

## Footnotes

1
www.hmpdacc.org

2
https://github.com/najoshi/sickle

3
https://bedtools.readthedocs.io/
