# Supplementary material for: Abundance of Lipopolysaccharide Heptosyltransferase I in Human Gut Microbiome and Its Association With Cardiovascular Disease and Liver Cirrhosis
Source: Front Microbiol. 2021 Nov 30;12:756976. doi: 10.3389/fmicb.2021.756976 (PMC8669917; doi:10.3389/fmicb.2021.756976)
Supplement: Supplementary file 2 [file Data_Sheet_1.PDF]

Supplementary Figure 1

A

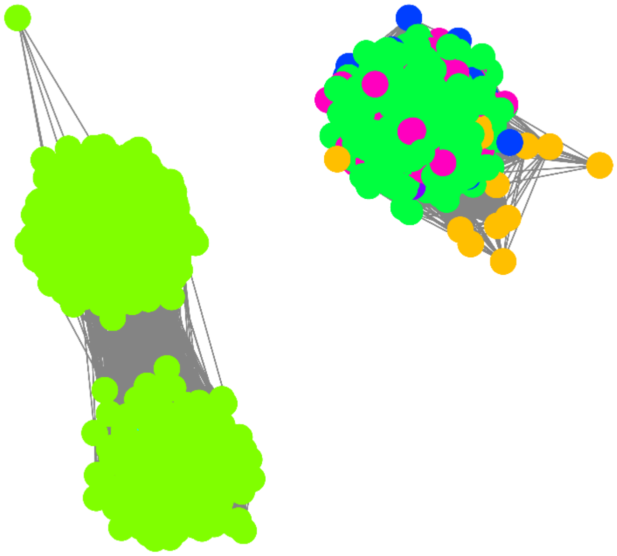

B

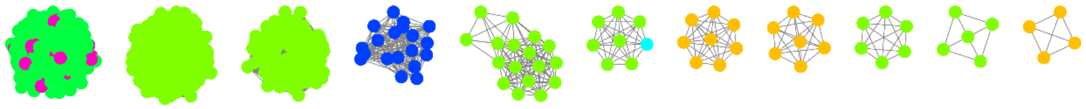

Supplementary Figure 1. HepI from the human gut microbiome analyzed to create the network (A sequence identity>40%; B sequence identity>45%).

Supplementary Figure 2

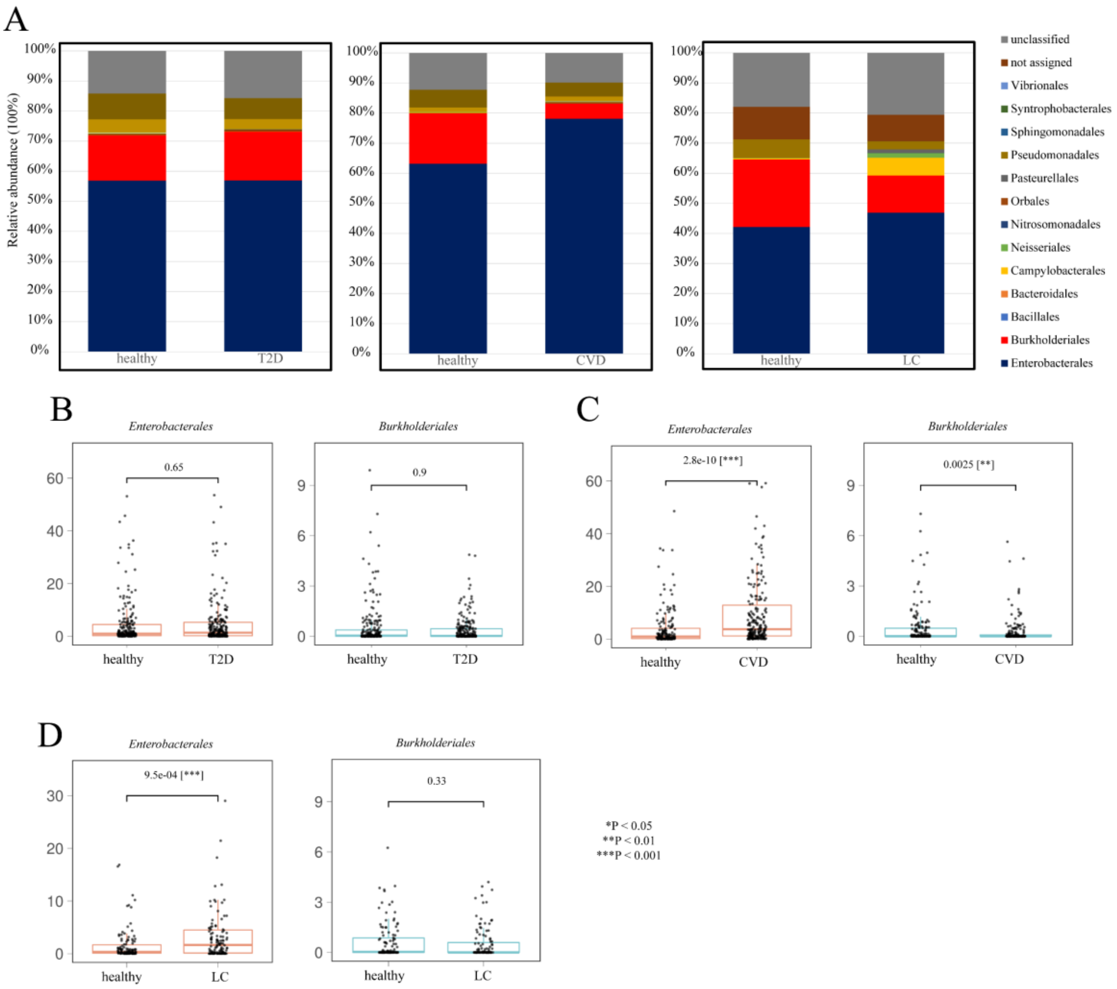

Supplementary Figure 2. Classification at the order level of HpeI and relative abundance of bacteria with HpeI in the healthy and disease conditions. (A) Taxonomic distribution of bacteria with HpeI at the order level in subjects of healthy and the disease conditions; Type 2 diabetes (T2D), cardiovascular disease (CVD), and liver cirrhosis (LC). The abundance of the bacteria with HpeIs from Enterobacterales and Burkholderiales was further compared as the healthy versus disease cohorts (T2D, B; CVD, C; and LC, D). Statistical significance was calculated using the paired Wilcoxon test. The p value was shown above in the figure.

Supplementary Table 1. The metagenomic sequencing datasets used in this study.

Accession number (BioProject)	Accession Number (SRA Study)	Number of contorl & case(s)	Nationality	Age (average±S.D.)	Gender Female (%)/Male (%)	Read number	Abbre.
PRJEB6337	ERP005860	114 (healthy) & 123 (liver cirrhosis) = 237 (total)	China	46±10.9	34.2/65.8	12,235,656,260	LC
PRJEB21528	ERP023788	171 (healthy) & 214 (ACVD) = 385 (total)	China	61±9.8	41/59	20,801,326,700	ACVD
PRJNA422434	SRP008047	healthy (183) & T2D (187) = total (370)	China	48±14.3	42.4/57.6	15,086,669,032	T2D
PRJEB1786	ERP002469	healthy (43) & T2D (53) = total (96)	Sweden	70±0.1	100/0	4,494,103,168	T2D
